# Knocking-out the Siah2 E3 ubiquitin ligase prevents mitochondrial NCX3 degradation, regulates mitochondrial fission and fusion, and restores mitochondrial function in hypoxic neurons

**DOI:** 10.1186/s12964-020-0529-x

**Published:** 2020-03-12

**Authors:** Maria Josè Sisalli, Gaetano Ianniello, Claudia Savoia, Ornella Cuomo, Lucio Annunziato, Antonella Scorziello

**Affiliations:** 1 Division of Pharmacology, Department of Neuroscience, Reproductive and Dentistry Sciences, School of Medicine, Federico II University of Naples, Via S. Pansini 5, 80131 Naples, Italy; 2 IRCCS SDN, Naples, Italy

**Keywords:** Mitochondria, siah2, NCX3, cortical neurons, Hypoxia

## Abstract

**Background:**

Na^+^/Ca^2^^+^ exchanger isoform 3 (NCX3) regulates mitochondrial Ca^2+^ handling through the outer mitochondrial membrane (OMM) and promotes neuronal survival during oxygen and glucose deprivation (OGD). Conversely, Seven In-Absentia Homolog 2 (Siah2), an E3-ubiquitin ligase, which is activated under hypoxic conditions, causes proteolysis of mitochondrial and cellular proteins.

In the present study, we investigated whether siah2, upon its activation during hypoxia, interacts with NCX3 and whether such interaction could regulate the molecular events underlying changes in mitochondrial morphology, i.e., fusion and fission, and function, in neurons exposed to anoxia and anoxia/reoxygenation.

**Methods:**

To answer these questions, after exposing cortical neurons from siah2 KO mice (siah2 −/−) to OGD and OGD/Reoxygenation, we monitored the changes in mitochondrial fusion and fission protein expression, mitochondrial membrane potential (ΔΨm), and mitochondrial calcium concentration ([Ca^2+^]_m_) by using specific fluorescent probes, confocal microscopy, and Western Blot analysis.

**Results:**

As opposed to congenic wild-type neurons, in neurons from siah2−/− mice exposed to OGD, form factor (FF), an index of the complexity and branching aspect of mitochondria, and aspect ratio (AR), an index reflecting the “length-to-width ratio” of mitochondria, maintained low expression. In KO siah2 neurons exposed to OGD, downregulation of mitofusin 1 (Mfn1), a protein involved in mitochondrial fusion and upregulation of dynamin-related protein 1 (Drp1), a protein involved in the mitochondrial fission, were prevented. Furthermore, under OGD conditions, whereas [Ca^2+^]_m_ was reduced, ΔΨm, mitochondrial oxidative capacity and ATP production were improved. Interestingly, our immunoprecipitation assay revealed that Siah2 interacted with NCX3. Indeed, siah2 knock-out prevented NCX3 degradation in neurons exposed to OGD. Finally, when siah2−/− neurons were exposed to OGD/reoxygenation, FF, AR, and Mfn1 expression increased, and mitochondrial function improved compared to siah2+/+ neurons.

**Conclusions:**

Collectively, these findings indicate that hypoxia-induced SIAH2-E3 ligase activation influences mitochondrial fusion and fission, as well as function, by inducing NCX3 degradation.

Video Abstract

## Background

We have recently showed that mitochondrial NCX3 (mNCX3) on the outer mitochondrial membrane forms a stable complex with the PKA anchoring protein AKAP121 and promotes mitochondrial calcium extrusion under physiological conditions. However, during hypoxia, mNCX3 activity is impaired and neuronal survival is compromised [1]. On the other hand the E3 ubiquitin ligase siah2 [2], which is activated under hypoxic conditions [3], translocates to the outer mitochondrial membrane and promotes AKAP121 proteolytic degradation, thereby causing mitochondrial dysfunction and oxidative stress.

Burgeoning evidence indicates that mitochondrial dysfunction is considered a key factor in triggering early neurodegenerative events [4–8]. Indeed, mitochondria play a crucial role in meeting the high metabolic demand of neurons by maintaining a constant energy supply through oxidative phosphorylation [9]. In addition to energy production, mitochondria are essential for regulating several processes necessary for neuronal functions, including intracellular calcium homeostasis, production of reactive oxygen species (ROS), apoptotic signaling, and synaptic function [10, 11]. The maintenance of mitochondrial function is strictly dependent on mitochondrial morphology, which is in turn regulated by the balance between fission and fusion [9, 12–14]. Indeed, an excess or a deficiency in either fission or fusion can have detrimental effects on the healthy population of mitochondria in neurons [12, 13, 15–17]. Fusion and fission are tightly regulated by a family of GTPase proteins. In particular, mitofusin 1 (Mfn1), mitofusin 2 (Mfn2), and OPA1, which are localized to the mitochondrial outer membrane [18, 19] and to the intermembrane space, respectively, regulate fusion processes. Instead, the dynamin-1-like protein (Drp1) [20–24], which is localized to the cytosol, regulates mitochondrial fission. Their overall activity is modulated by phosphorylation, sumoylation, and ubiquitylation [25–31].

In this regard, studies show that Drp1 is a direct substrate of PKA. PKA activation increases Drp1 phosphorylation, thereby inhibiting its enzymatic activity [27] and preventing mitochondrial fission. On the other hand, intracellular events such as membrane depolarization, which promotes Drp1 dephosphorylation, causes mitochondrial fission and cell death [26].

Here we examined whether Siah2 activation in neurons exposed to hypoxia might interact with mNCX3, thus altering mitochondrial calcium efflux and whether such interaction might participate in the molecular events regulating mitochondrial fission and fusion.

Thus, we exposed cortical neurons from siah2 KO (siah2 −/−) and WT mice (siah2+/+) to OGD and OGD/Reoxygenation and observed changes in mitochondrial fusion and fission by using specific fluorescent probes, confocal microscopy, and mitochondrial proteins expression. Moreover, we assessed mitochondrial function by measuring mitochondrial membrane potential (ΔΨ_m_), mitochondrial calcium concentration ([Ca^2+^]_m_), and ATP production.

## Methods

### Cell culture

Primary cortical neurons from siah2+/+ and siah2−/− mice were obtained from 15 to 16 day old embryos as previously reported [32]. After removal of the brain cortices were isolated and subjected to mechanical and enzymatic digestion for 30 min in the presence of trypsin/EDTA at 37 °C. After incubation, the tissues were centrifuged (2500 rpm, 5 min), the supernatant was removed, and the pellet was resuspended in MEM/F12 culture medium (Life technologies) containing: horse serum (5%), fetal bovine serum (5%), glucose (30%), and antibiotics (penicillin/streptomicyn 0,5%). Neurons were then plated either in plastic petri-dishes and in 12 multiwell dishes, for Western Blot and MTT experiments, respectively, or on 25 mm quartz cover slips for confocal experiments. Neurons were maintained at 37 °C in a humidified atmosphere of 5% CO_2_ and 95% air and used after 10 days.

### Combined oxygen and glucose deprivation (OGD) and reoxygenation (REOXY)

Cortical neurons were first exposed to OGD for 3 h and then to reoxygenation for 24 h [33, 34]. In brief, the culture medium was replaced with a hypoxia medium, which was previously saturated with 95% N_2_ and 5% CO_2_ for 20 min; it contained NaCl 116 mM, KCl 5.4 mM, MgSO_4_ 0.8 mM, NaHCO_3_ 26.2 mM, NaH_2_PO_4_ 1 mM, CaCl_2_ 1.8 mM, glycine 0.01 mM, and 0.001 w/v phenol red. Hypoxic conditions were maintained with a hypoxia chamber (temperature 37 °C, atmosphere 95% N_2_ and 5% CO_2_). These experimental conditions induced a 30% decrease of pO_2_ in the medium.

Deprivation of oxygen and glucose was stopped by placing the cells in the regular culture medium saturated with a mixture of 95% O_2_ and 5% CO_2_ for 10 min. Reoxygenation was achieved by returning neurons to normoxic conditions (37 °C in a humidified 5% CO_2_ atmosphere) for 24 h.

MG132 (40 μM) was added to the hypoxic medium during OGD.

### Transient ischemia

Transient focal ischemia was induced, as previously described [35], by suture occlusion of the right middle cerebral artery in male mice anesthetized with 1.5% sevoflurane, 70% N_2_O, and 28.5% O_2_. Achievement of ischemia was confirmed by monitoring regional cerebral blood flow in the area of the right middle cerebral artery through a laser Doppler (PF5001; Perimed, Sweden). Animals not showing a cerebral blood flow reduction of at least 70% (*n* = 8), as well as those dying after ischemia induction (*n* = 5), were excluded from the study. Rectal temperature was maintained at 37 ± 0.5 °C with a thermostatically controlled heating pad and lamp. After 60 min of middle cerebral artery occlusion, mice were reanesthetized and the filament was withdrawn to restore blood flow. Animals were randomized into the different experimental groups, containing each one 5 to 8 animals, in a blind manner. Sham-operated animals were subjected to the entire surgical procedure for tMCAO induction except for the insertion of the filament.

Mg132 (1 μl) was icv administered from a 40 mM stock twice, 3 h before the induction of transient ischemia and immediately after filament withdrawn. In particular, anesthetized mice were positioned on a stereotaxic apparatus and a cannula (Plastic one) was implanted into the right lateral ventricle using the stereotaxic coordinates from the bregma: 0.4 mm caudal, 1.2 mm lateral, and 2 mm below the dura and secured to the skull with dental cement [35].

### Analysis of mitochondrial morphology using the IMAGEJ 1.42 software

Mitochondria were labeled by incubating cells with MitoTracker Red (Invitrogen, 20 nM) for 20 min prior to acquisition. Digital images were captured on a confocal microscope, with a 63X oil immersion lens and subjected to a 2D deconvolution step, which is meant to compensate for various optical imperfections. Accordingly, the “Interative Deconvolution”, a plug-in written by Bob Dougherty for ImageJ was used. After image enhancements, mitochondrial shape metrics were reported by an ImageJ macro, “Morphometry”, described by Cribbs and Strack [36]. This macro allowed us to determine two parameters of mitochondrial morphology: form factor (FF) and aspect ratio (AR). The former takes into account the perimeter and area of a single mitochondrion and can therefore capture complex mitochondrial shapes. The latter, instead, despite being a useful shape metric for simple rod-like mitochondria, does not faithfully represent the shape of linked, branched, or highly interconnected mitochondria. Low values of FF and AR indicate circular mitochondria, whereas high values indicate elongated and highly interconnected mitochondria [36].

### Western blot

Protein samples (50 μg) were analyzed on 8% (NCX3) or 10% (Drp1, Mfn1 and Mfn2) sodium dodecyl sulfate polyacrilamide gel with 5% sodium dodecyl sulfate stacking gel (SDS-PAGE) and electrotransferred onto Hybond ECL nitrocellulose paper (Amersham) [37]. Membranes were blocked with 5% non fat dry milk in 0.1% Tween-20 (TBS-T; 2 mM Tris–HCl, 50 mM NaCl, pH 7.5) for 2 h at RT and subsequently incubated overnight at 4 °C in the blocked buffer with antibodies for NCX3 (polyclonal rabbit antibody, Philipson’s Laboratory; 1:5000), Mfn1 (Millipore, 1:1000), Drp1 (BD biosciences, 1:1000), VDAC (Millipore 1:1000), Mfn2 (Millipore, 1:1000), tubulin (Sigma-Aldrich, 1:10000), and actin (Sigma-Aldrich, 1:1000). The membranes were washed with 0.1% Tween 20 and incubated with secondary antibodies for 1 h (1,1000; Amersham). Immunoreactive bands were detected with ECL (Amersham).

### Imaging of mitochondrial Ca^2+^ and mitochondrial membrane potential

[Ca^2+^]_m_ was assessed by using the fluorescent dye X-Rhod1. In brief, cells were loaded with X-Rhod1 0.2 μM for 15 min in a medium containing 156 mM NaCl, 3 mM KCl, 2 mM MgSO_4_, 1.25 mM KH_2_PO_4_, 2 mM CaCl_2_, 10 mM glucose, and 10 mM Hepes. The pH was adjusted to 7.35 with NaOH. After incubation, cells were washed 3 times in the same medium. An increase in mitochondria-localized intensity of fluorescence suggested mitochondrial Ca^2+^ overload [37].

Mitochondrial membrane potential was assessed by using the fluorescent dye tetramethyl rhodamine ethyl ester (TMRE) in the “redistribution mode”. Cells were loaded with TMRE (20 nM) for 30 min in the above described medium. At the end of the incubation, the cells were washed in the same medium containing TMRE (20 nM) and allowed to equilibrate. A decline in mitochondria-localized intensity of fluorescence was indicative of mitochondrial membrane depolarization.

Confocal images were obtained with a Zeiss inverted 700 confocal laser scanning microscopy and a 63X oil immersion objective. The illumination intensity of 543 Xenon laser, used to excite X-Rhod-1 and TMRE fluorescence, was kept to a minimum of 0.5% of laser output to avoid phototoxicity [38].

### ATP detection

ATP content was measured with a commercial bioluminescent assay (ATP bioluminescent assay kit, Sigma, St. Louis, Missouri, USA) according to the manufacturer’s instruction. In brief, ATP was extracted by boiling the samples in a solution containing (in mM) 100 TRIS, 4 EDTA, pH 7.75, and then centrifuged at 10,000×g for 60s**.** To obtain bioluminescence measurements with a standard luminometer, 100 μL of supernatant was mixed with 100 μL of luciferin–luciferase solution. The standard curve of ATP was obtained by serial dilution of 2 μM ATP solution [39].

### MTT assay

Mitochondrial activity was assessed by 3-(4,5-dimethylthiazol-2-yl)-2,5, diphenyltetrazolium bromide (MTT) assay as previously described [34, 37]. The assay was based on the red-ox ability of living mitochondria to convert dissolved MTT into insoluble formazan. In brief after treatments, the medium was removed and cells were incubated in 1 ml of MTT solution (0.5 mg/ ml) for 1 h in a humidified 5% CO_2_ incubator at 37 °C. To stop incubation, MTT solution was removed and 1 ml dimethyl sulfoxide was added to solubilize the formazan product. The absorbance was monitored at 540 nm with a Perkin-Elmer LS 55 luminescence spectrometer (Perkin-Elmer Ltd., Beaconsfield, England). The data are expressed as percentage of cell damage compared with sham-treated cultures.

### Immunoprecipitation and immunoblot analyses

Cortical neurons and brain cortex from siah2+/+ control mice and mice exposed to tMCAO were homogenized in lysis buffer containing 50 mM Tris-HCl pH 7.4, 0.15 M NaCl, 1 mM EDTA, 1% Triton X-100, 100 mM NaF, 100 mM Na_3_VO_4_, 5 μg/ml aprotinin, 10 μg/ml leupeptin**,** and 2 μg/ml pepstatin. One milligram of lysate was precleared using protein A/Gplus (Santa Cruz, Dallas, TX) for 1 h at 4 °C with constant rotation and centrifuged for 2 min at 8000 rpm. The cell lysates (1 mg) were cleared by centrifugation at 15,000 x g for 15 min and were immunoprecipitated with anti-Siah2 mouse antibody (Sigma-Aldrich, 1:100). An aliquot of cell lysate (100 μg) or immunoprecipitates were resolved by SDS-PAGE gel and transferred onto nitrocellulose membrane. Immunoblot analysis was performed using anti NCX3 and VDAC antibodies, as previously described [1]. Chemio-luminescent (ECL) signals were quantified by Chemi Doc Imaging System (Biorad).

Regarding lysates from brain tissue, 1 mg of precleared lysate was immunoprecipitated with an anti-SIAH2 mouse antibody (Sigma-Aldrich, 1:100) using the same experimental procedure described above. Finally, total lysates or immunoprecipitates were resolved by SDS-PAGE gel and transferred to a nitrocellulose membrane. Immunoblot analysis was performed using anti-NCX3 and anti-VDAC antibodies.

### Statistical analysis

Data were generated from a minimum of three independent experiments. Calcium measurements were performed in at least 20 cells for each independent experiments. Data are expressed as mean + S.E.M. Statistical analysis was performed with analysis of variance followed by Newman-Keuls test. Statistical significance was accepted at the 95% confidence level (*P* + 0.05).

## Results

### *siah2* gene ablation prevents mitochondrial fragmentation and hypoxia-induced ncx3 degradation, thereby preserving mitochondrial function in primary cortical neurons exposed to OGD and OGD/Reoxygenation

**Fig. 1:**
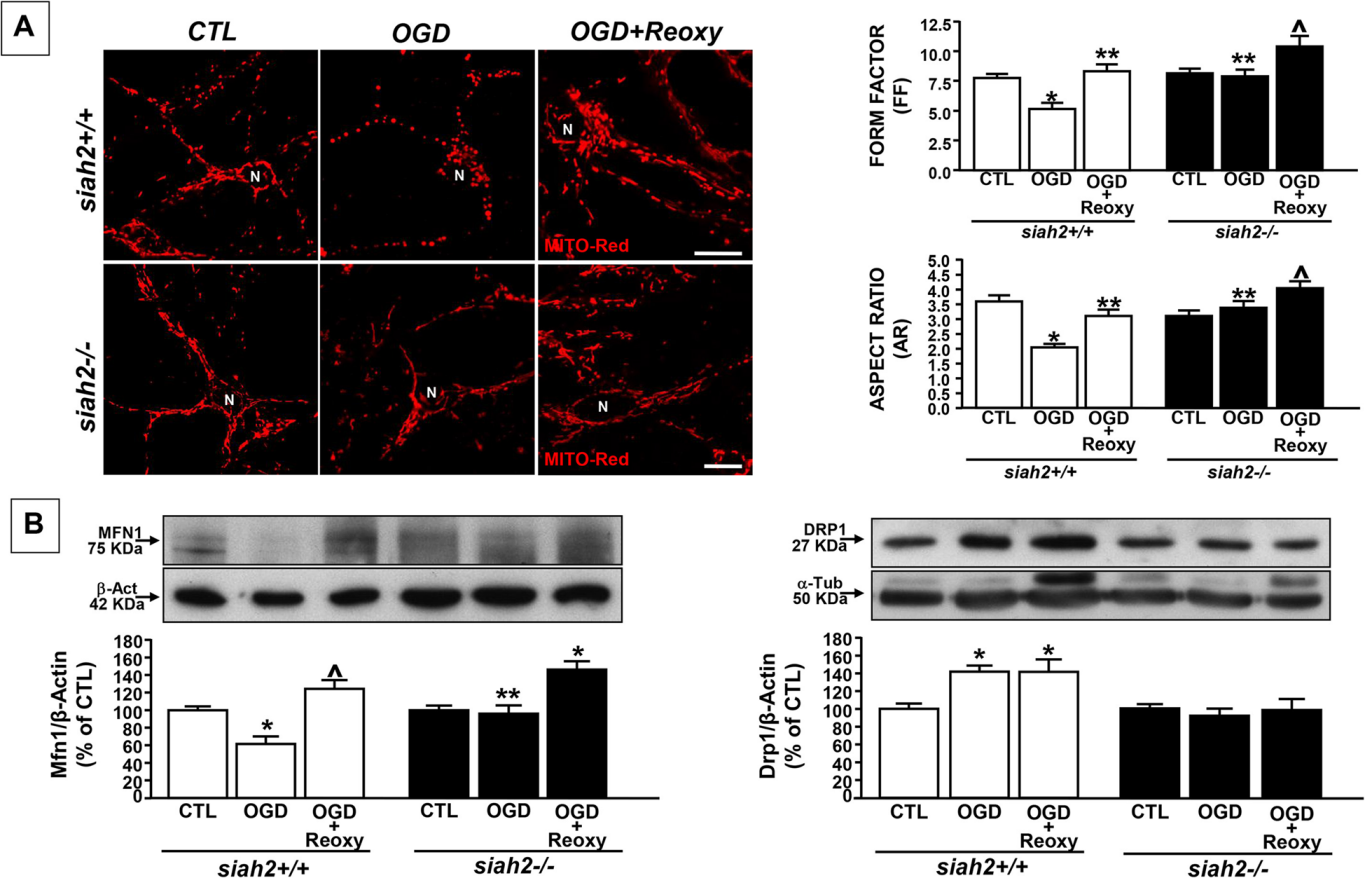
Mitochondrial morphology in primary cortical neurons obtained from siah2+/+ and siah2−/− mice exposed to OGD and OGD/Reoxygenation. (**a**- left) Imaging mitochondrial morphology in siah+/+ and siah−/− cortical neurons by confocal microscopy and MitoTracker Red (20 nM) (left panel). N: neurons, scale bars: 10 μm. (**a**- right) quantification of the changes in mitochondrial morphology by Image J software. Form factor (FF) and Aspect ratio (AR) in siah+/+ and siah−/− neurons. **b** Western Blot analysis of Mfn1 and DRP1 protein expression in siah2+/+ and siah2−/−cortical neurons exposed to OGD and OGD/Reoxygenation. Each bar represents the mean + S.E.M. of the percentage of different experimental values obtained in three independent experimental sessions. *P < 0.05 vs siah2+/+ CTL; **P < 0.05 vs siah2+/+ OGD; ^*P* < 0.05 vs siah2+/+ OGD/Reoxy

**Fig. 2:**
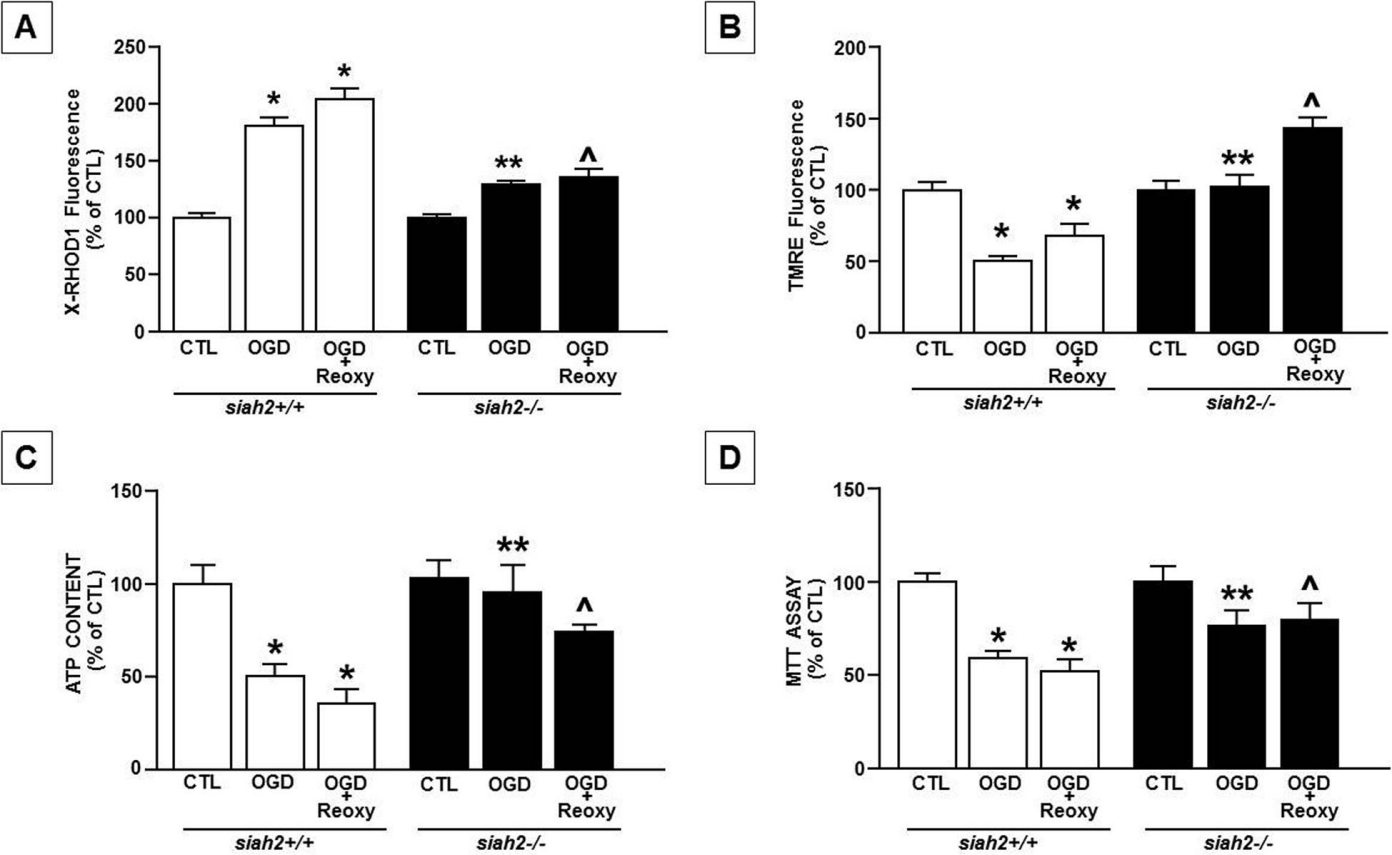
Mitochondrial function in primary cortical neurons obtained from siah2+/+ and siah2−/− mice exposed to OGD and OGD/Reoxygenation. Quantification of mitochondrial parameters in primary cortical neurons obtained from siah2+/+ and siah2−/− mice exposed to OGD and OGD followed by reoxygenation: **a** Mitochondrial calcium concentration, **b** Mitochondrial membrane potential, **c** Mitochondrial oxidative capacity and **d** ATP production. Each bar represents the mean + S.E.M. of the percentage values of at least 20–30 neurons recorded in three independent experimental sessions. *P < 0.05 vs siah2+/+ CTL; **P < 0.05 vs siah2+/+ OGD; ^*P* < 0.05 vs siah2+/+ OGD/Reoxy

Whereas exposure of siah2+/+ neurons to OGD reduced form factor (FF) and aspect ratio (AR), it did not in siah2−/− neurons (Fig. 1a). Moreover, exposure of siah2+/+ neurons to OGD increased Drp1 expression and reduced Mfn1 (Fig. 1b). Such changes were counteracted by siah2 ablation (Fig. 1 a-b). Regarding mitochondrial function, exposure of siah2+/+ neurons to OGD led to an increase in mitochondrial calcium, mitochondrial membrane depolarization, ATP reduction**,** and mitochondrial oxidative damage (Fig. 2).

**Fig. 3:**
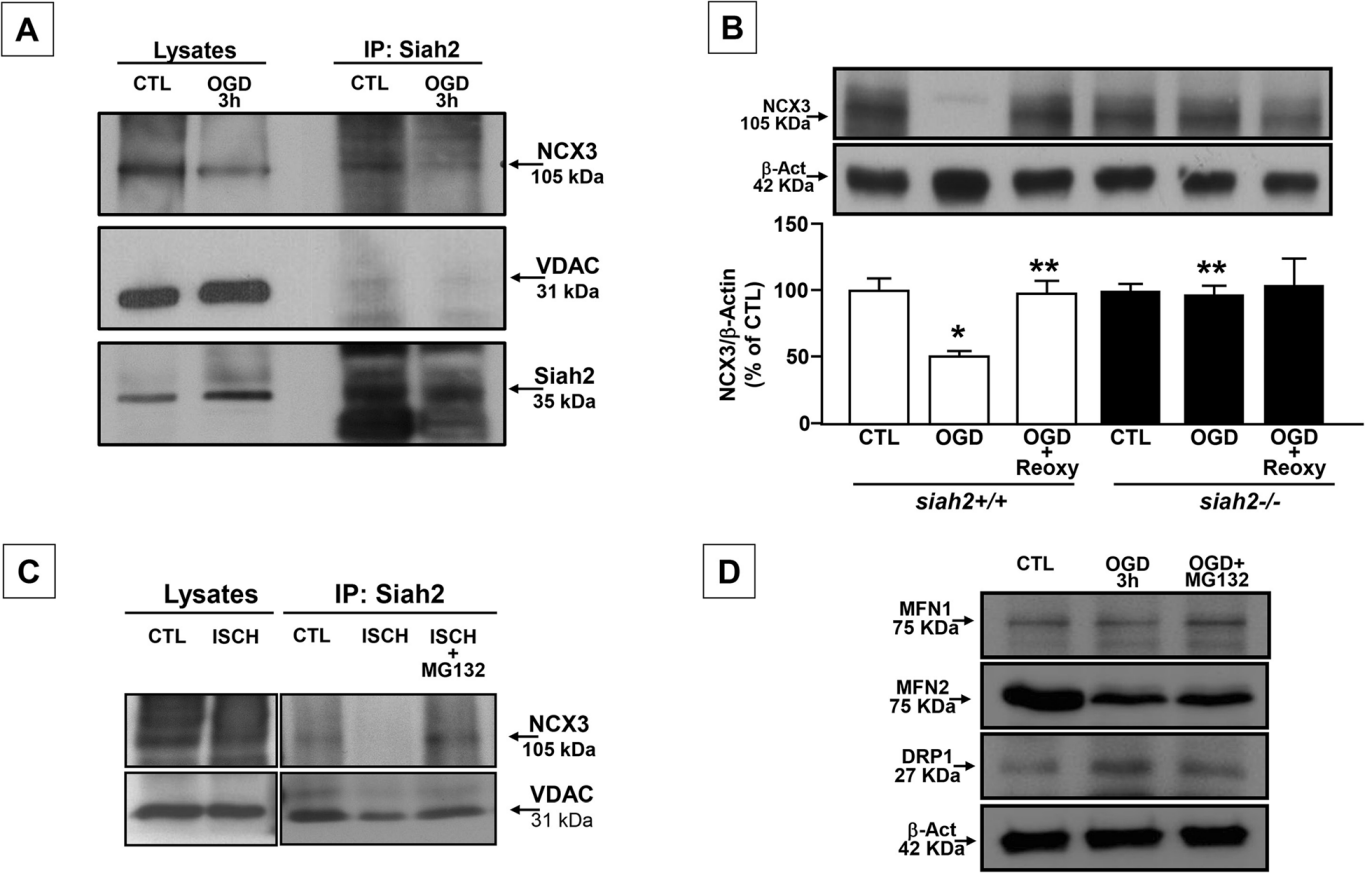
Biochemical interaction between SIAH2 and NCX3. **a** Immunoprecipitation assay in siah2+/+ neurons. **b** NCX3 protein expression in siah2+/+ and siah2−/− cortical neurons exposed to OGD and OGD/Reoxygenation. **c** Immunoprecipitation assay in siah2+/+ mice treated with the proteasome inhibitor MG132 (1 μl was administered icv from a 40 mM stock twice, 3 h before the induction of transient ischemia and immediately after filament withdrawn). **d** Mfn1, Mfn2, and Drp1 protein expression in siah2+/+ neurons exposed to OGD in the presence and in the absence of MG132 (40 μM). Each bar represents the mean +/– S.E.M. of the percentage of different experimental values obtained in three independent experimental sessions. *P < 0.05 vs siah2+/+ CTL; **P < 0.05 vs siah2+/+ OGD; ^*P* < 0.05 vs siah2+/+ OGD/Reoxy

By contrast, exposure of siah2−/− neurons to OGD decreased [Ca^2+^]_m,_ hyperpolarized the mitochondrial membrane, and preserved both mitochondrial ATP production and oxidative capacity (Fig. 2). Interestingly, siah2 ablation prevented downregulation of NCX3 protein expression observed in siah2+/+ neurons exposed to OGD (Fig. 3a). Moreover, the exposure of siah2+/+ neurons to OGD induced an impairment of Mfn2 expression that was reverted by the proteasome inhibitor MG132 (Fig. 3d). Similarly, the exposure of siah2+/+ neurons to OGD in the presence of MG132 restored the level of Mfn1, and did not affect Drp1 increase observed during OGD (Fig. 3d).

However, exposure of siah2+/+ and siah2−/− neurons to OGD/Reoxygenation increased FF and AR compared to controls (Fig. 1a). This effect was accompanied by a rise in Mfn1 expression, (Fig. 1b left), albeit more pronounced in siah2−/− neurons.

Conversely, OGD followed by reoxygenation did not affect Drp1 protein expression. Indeed, in both siah2+/+ and siah2−/− neurons, its levels were similar to those detected during OGD (Fig. 1b right). Interestingly, in siah2+/+ and siah2−/− neurons exposed to OGD followed by reoxygenation, NCX3 protein expression levels were comparable to those of controls (Fig. 3b). However, OGD plus reoxygenation increased [Ca^2+^]_m_, depolarized mitochondrial membrane, and impaired ATP production and oxidative capacity in siah2+/+ neurons. (Fig. 2a-d). Conversely, after reoxygenation whereas [Ca^2+^]_m_ was damaged and the mitochondrial membrane was hyperpolarized, ATP production and mitochondrial oxidative activity were preserved in siah2−/− neurons (Fig. 2a-d).

### SIAH2 immunoprecipitates with NCX3 and induces its degradation in cortical neurons exposed to OGD and in siah2+/+ mice exposed to tMCAO

To verify whether NCX3 interacts with SIAH2, siah2+/+ neurons were immunoprecipitated under normoxic and hypoxic conditions. Immunoprecipitation with selective SIAH2 antibody detected NCX3 on the outer mitochondrial membrane, as demonstrated by the presence of the specific marker VDAC in the immunoprecipitate (Fig. 3 a). This phenomenon was observed under both normoxic and OGD conditions. Similarly, immunoprecipitation experiments with selective SIAH2 antibody performed in siah2+/+ mice, demonstrated a direct binding between NCX3 and SIAH2 at mitochondrial level, as demonstrated by VDAC positivity in the immunoprecipitate (Fig. 3c). Interestingly, in siah2 +/+ mice exposed to tMCAO the immunoprecipitation between NCX3 and SIAH2 was absent whereas the treatment of the animals with MG132 restored this physical interaction, thus suggesting that the activation of SIAH2 induced by ischemic insult might be responsible for NCX3 protein degradation observed in hypoxic conditions.

### *ncx3* gene ablation induces mitochondrial dysfunction and causes mitochondrial fragmentation in cortical neurons

**Fig. 4:**
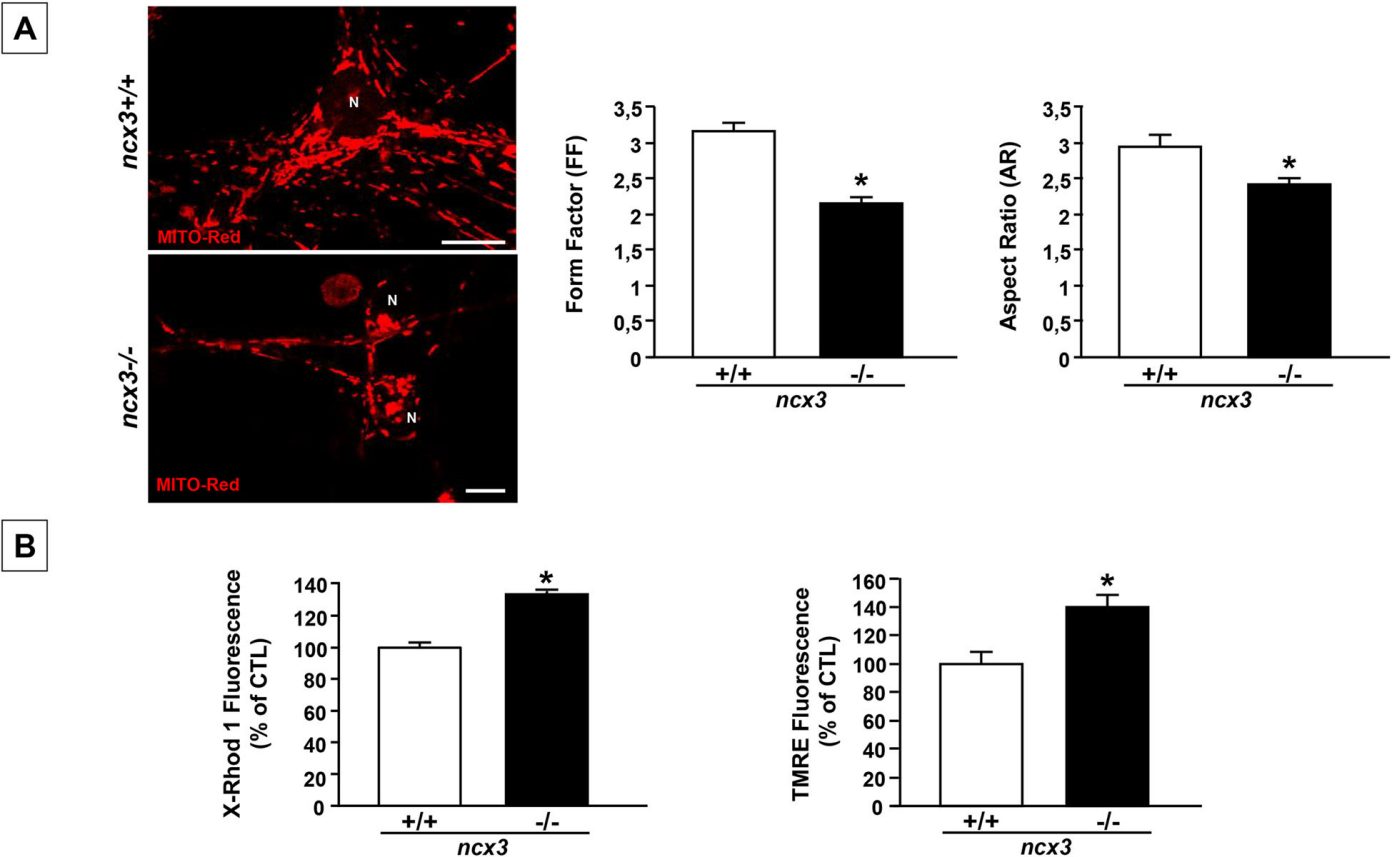
Mitochondrial morphology and function in primary cortical neurons obtained from ncx3+/+ and ncx3−/− mice. (**a**-left), Imaging mitochondrial morphology in ncx3+/+ and ncx3−/− cortical neurons by confocal microscopy and MitoTracker Red (20 nM), N: neurons; scale bars: 10 μm. (**a**-right), quantification of the changes in mitochondrial morphology by Image J software. Form Factor (FF) and Aspect ratio (AR) in ncx3−/− neurons. **b** Confocal analysis of mitochondrial membrane potential and mitochondrial calcium concentration in ncx3+/+ and ncx3−/− cortical neurons. Each bar represents the mean + S.E.M. of the percentage of different experimental values obtained in three independent experimental sessions. *P < 0.05 vs ncx3+/+ and CTL; **P < 0.05 vs OGD

To confirm that NCX3 plays a key role in regulating mitochondrial morphology, further experiments were performed in cortical neurons obtained from ncx3−/− mice. As opposed to wild-type neurons, cortical neurons from ncx3−/− displayed changes in mitochondrial morphology, as confirmed by the reduced levels of FF and AR (Fig. 4a). Moreover, these morphological changes were accompanied by mitochondrial membrane hyperpolarization and by increased levels of x-Rhod1-monitored mitochondrial calcium (Fig. 4b).

## Discussion

This study demonstrates that OGD-induced hypoxia triggers mitochondrial fragmentation (fission) and reduces fusion, as evidenced by increases in Drp1 and decreases in Mfn1 and Mfn2. These morphological changes were accompanied by increases in [Ca^2+^]_m_ and impairment in mitochondrial membrane potential. Moreover, activation of the E3 ubiquitin ligase SIAH2 impaired mitochondrial integrity by promoting proteolytic degradation of NCX3 and consequent Ca^2+^ overload into mitochondria during OGD and reoxygenation.

In addition, we evidenced that there is a tight correlation between mitochondrial dysfunction and mitochondrial morphological changes. Accordingly, we hypothesize that mitochondrial fragmentation occurs in response to the metabolic impairment arising under hypoxic conditions. Consistently, Shutt and McBride showed that when mitochondria are depolarized they undergo fragmentation, which, in turn, triggers the clearance of these organelles through an autophagic pathway [40]. Such finding suggests that mitochondrial fission and fusion might be part of a more complex mechanism aimed at activating the “mitochondrial quality control” system every time a stressful condition impairs mitochondrial function [40]. Indeed, in the present work, siah2 ablation, by preventing NCX3 degradation elicited by OGD, was able to preserve the balance between fragmentation and fusion, and to counteract increases in [Ca^2+^]_m_ and mitochondrial depolarization. These new findings extend our previous data showing that the PKA mitochondrial anchoring protein AKAP121 [2], which operates as an interactor of NCX3 on the outer mitochondrial membrane [1], is another proteolytic target of SIAH2 as already reported for AKAP121 by Ronai’s Lab in 2011 [3]**.** This hypothesis is further supported by immunoprecipitation experiments in vitro and in vivo demonstrating a physical interaction between SIAH2 and NCX3. As consequence, during hypoxic/ischemic conditions the activation of SIAH2 leads to NCX3 proteolytic degradation prevented by MG132 treatment, thus confirming that the physical interaction between the two proteins is responsible for the impairment of NCX3 protein expression. Thus, on the basis of our present and previous studies, we hypothesize that NCX3, AKAP121, and SIAH2 constitute a crucial pathway for preserving mitochondrial function and for maintaining mitochondrial integrity during metabolic stress conditions such as hypoxia. As reported for other proteins [1, 41–46], we speculate that when SIAH2 is activated during hypoxia, it translocates to depolarized mitochondria where it induces the degradation of NCX3 and AKAP121 via the proteasome, similar to the behavior of other protein complexes including the Pink1/parkin complex in Parkinson’s disease [46–48].

On the other hand, AKAP121 also mediates mitochondrial morphology by modulating Drp1 activity and recycling [3, 49]. Indeed, AKAP121 inhibits Drp1 through PKA-dependent phosphorylation, thereby hampering Drp1-Fis1 interaction on the OMM [3, 49].

Furthermore, that NCX3 and AKAP121 are key proteins necessary to maintain mitochondrial morphology, owing to their ability to promote mitochondrial Ca^2+^ homeostasis, is testified by our experiments in neurons obtained from ncx3−/− mice. We found higher calcium levels and fragmentation in ncx3−/− neurons than in ncx3+/+ neurons a finding suggesting the role of NCX3 in regulating mitochondrial dynamics.

Another novel aspect of the present study is the finding that the lack of degradation of NCX3 and AKAP121 observed in siah2−/− neurons had a neuro-beneficial role not only during OGD but also during reoxygenation, as evidenced by the preservation of mitochondrial functional activity, as well as fusion and fission dynamics.

Conversely, although subsequent exposure of siah2+/+ neurons to reoxygenation, restored NCX3 and AKAP121 protein expression levels, it gave rise to a dysfunctional and elongated mitochondria. These findings led us to hypothesize that the activation of the SIAH2/NCX3/AKAP121 pathway during OGD is responsible for eliciting mitochondrial damage during the reoxygenation phase. Similarly, under conditions causing oxidative stress, as it occurs during the reoxygenation, mitochondria are elongated [50, 51]. Moreover, the results obtained during the reoxygenation phase allowed us to validate the hypothesis that the changes in mitochondrial morphology are strictly related to ATP production and cellular redox activity.

## Conclusions

Taken together our findings led us to conclude that during OGD and reoxygenation (1) the SIAH2/NCX3/AKAP121 pathway regulates fission and fusion balance by controlling mitochondrial morphology and mitochondrial oxidative metabolism; (2) AKAP121 works as an ancillary element in the SIAH2/NCX3/AKAP121 complex by interacting with NCX3 and SIAH2. Indeed, in cells devoid of NCX3 activity the function and morphology of mitochondria are both compromised; (3) the inhibition of SIAH2 might be explored as a new druggable target to prevent mitochondrial damage. Indeed, its ablation is able to preserve mitochondrial function and morphology under ischemic conditions.

## Data Availability

All raw data are available on request.

## Abbreviations

[Ca^2+^]m: Mitochondrial calcium concentration
AKAP121: PKA anchoring protein
AR: Aspect ratio
Drp1: Dynamin-related protein 1
FF: Form factor
Mfn1: Mitofusin 1
Mfn2: Mitofusin 2
mNCX3: Mitochondrial Na+/Ca + exchanger isoform 3
MTT: 3-(4,5-dimethylthiazol-2-yl)-2,5, diphenyltetrazolium bromide
NCX3: Na+/Ca + exchanger isoform 3
OGD: Oxygen and glucose deprivation
OMM: Outer mitochondrial membrane
OPA1: Mitochondrial dynamin like GTPase
PKA: Protein kinase A
ROS: Oxygen species reactive
Siah2: Seven In-Absentia Homolog 2
tMCAO: Transient Middle Cerebral Artery Occlusion
TMRE: Tetramethyl rhodamine ethyl ester
VDAC: Voltage-dependent anion channel
ΔΨm: Mitochondrial membrane potential
